# Fluticasone/salmeterol reduces remodelling and neutrophilic inflammation in severe equine asthma

**DOI:** 10.1038/s41598-017-09414-8

**Published:** 2017-08-18

**Authors:** Michela Bullone, Amandine Vargas, Yvonne Elce, James G. Martin, Jean-Pierre Lavoie

**Affiliations:** 1 Université de Montréal, Faculty of Veterinary Medicine, Department of Clinical Sciences, 3200 rue Sicotte, St-Hyacinthe, J2S 2M2 Quebec, Canada; 2 McGill University, Meakins Christie Laboratories, McGill University Health Center Research Institute, 1001 Decarie Blvd, Montreal, H4A 3J1 Quebec, Canada; 3 University of Edinburgh, Royal (Dick) School of Veterinary Studies, Easter Bush Campus, EH25 9RG United Kingdom

## Abstract

Asthmatic airways are inflamed and undergo remodelling. Inhaled corticosteroids and long-acting β2-agonist combinations are more effective than inhaled corticosteroid monotherapy in controlling disease exacerbations, but their effect on airway remodelling and inflammation remains ill-defined. This study evaluates the contribution of inhaled fluticasone and salmeterol, alone or combined, to the reversal of bronchial remodelling and inflammation. Severely asthmatic horses (6 horses/group) were treated with fluticasone, salmeterol, fluticasone/salmeterol, or with antigen avoidance for 12 weeks. Lung function, central and peripheral airway remodelling, and bronchoalveolar inflammation were assessed. Fluticasone/salmeterol and fluticasone monotherapy decreased peripheral airway smooth muscle remodelling after 12 weeks (p = 0.007 and p = 0.02, respectively). On average, a 30% decrease was observed with both treatments. In central airways, fluticasone/salmeterol reversed extracellular matrix remodelling after 12 weeks, both within the lamina propria (decreased thickness, p = 0.005) and within the smooth muscle layer (p = 0.004). Only fluticasone/salmeterol decreased bronchoalveolar neutrophilia (p = 0.03) to the same extent as antigen avoidance already after 8 weeks. In conclusion, this study shows that fluticasone/salmeterol combination decreases extracellular matrix remodelling in central airways and intraluminal neutrophilia. Fluticasone/salmeterol and fluticasone monotherapy equally reverse peripheral airway smooth muscle remodelling.

## Introduction

Bronchial wall remodelling is a hallmark of asthma resulting from chronic inflammation and bronchospasm-induced mechanical stress^1, 2^. Airway smooth muscle (ASM), whose increase is proportional to disease severity^3^ and caused by cellular hyperplasia and hypertrophy^4^, is the major contributor to airway obstruction^5^. Increased deposition of extracellular matrix (ECM) elements is also a typical feature of severe asthma cases^6^. Multiple studies have focused on central ASM remodelling using samples obtained by endobronchial biopsy (EBB). However, EBBs provide partial-thickness samples limited to the carinae of the central airways^7^, while post-mortem studies on entire lungs of severe asthmatics have shown that the most marked alterations occur in the peripheral airways, that is, those <2 mm in diameter^3, 8^.

Peripheral airway dysfunction is associated with poor disease control and with neutrophilic inflammation in asthma^9, 10^. Combining anti-inflammatory and bronchodilating treatments has a synergistic effect on disease control even in severe asthmatic patients, which has been ascribed to an enhanced penetration within the bronchial tree or to their synergic anti-inflammatory effects^11^. However, disease control is usually expressed in terms of spirometry and patient-reported symptom perception tests, two unreliable indicators of peripheral airway dysfunction^12^. The effectiveness of currently employed asthma treatments at this level on remodelling and inflammation is thus questionable^13^. Long-term ICS monotherapy effectively reduces the number of peripheral lung cells expressing smooth muscle actin in severe asthmatic patients^14^, but a reduction of parenchymal myofibroblasts rather than ASM cells could have accounted for the difference observed. In the same study, long-term ICS monotherapy did not decrease the quantity of collagen in peripheral lung samples and a different regulation of remodelling reversal was reported centrally and peripherally. Ultimately, whether ICS/LABA combination can reverse established remodelling in peripheral asthmatic airways and whether this is reflected by significant changes in the central airways remains undetermined.

Indirect small airway assessment in asthma is limited by the paucity of diagnostic tools available^15^. Direct sampling is challenging and requires invasive procedures^16^, whose implementation in human studies is limited by ethical considerations. For these reasons, animal models represent essential tools for investigating small airway pathophysiology and response to treatment in asthma. Adult horses exposed to barn antigens can develop severe equine asthma (previously known as “heaves”), a disease characterized by airway remodelling and neutrophilic inflammation^17, 18^. Equine lung samples and endobronchial biopsies can be easily harvested, which makes asthmatic horses ideal models for the translational study of asthma-associated remodelling of the peripheral and central airways^17, 19^. This study was designed for evaluating the specific contribution of anti-inflammatory and bronchodilator treatments, alone or combined, to lung function, inflammation control, and remodelling reversal in the equine model of neutrophilic asthma. We hypothesized that combination therapy with ICS/LABA enhances peripheral remodelling reversal by enhanced anti-inflammatory effect.

## Results

### Animals

Table 1 reports physiological details of the horses studied. Haematological results obtained at baseline were within normal ranges, excluding the possibility of concomitant respiratory infections in the horses studied. In study I, 2/13 horses had slightly increased blood fibrinogen (5 g/L, reference range: 2–4 g/L). In study II, only 1 horse had fibrinogen at 5 g/L. Increased blood fibrinogen concentration is reported in asthmatic horses^20^.

**Table 1:** Horses’ description.

	Study I			Study II		
	Antigen avoidance	Fluticasone/Salmeterol	*p value*	Fluticasone	Salmeterol	*p value*
N	7*	6		6	6	
Age [years]	18.5 ± 6.3	15.3 ± 4.5	*0.36*	14.3 ± 5.6	15.2 ± 3.7	*0.66*
Weight [kg]	487 ± 91	544 ± 115	*0.34*	513 ± 57	543 ± 74	*0.82*
Sex [f/m]	4/3	5/1	*0.56*	5/1	3/3	*0.54*
Disease duration† [years]	4.6 ± 2.6	3.3 ± 1.4	*0.42*	4.5 ± 3.5	4.0 ± 1.3	*0.69*
Disease severity [R_L_ < 3 / R_L_ > 3]^¶^	3/4	5/1	*0.56*	3/3	3/3	*1*

Eight months elapsed between study I and II. Seven out of 17 horses participated in both studies, equally distributed between groups. *One horse in the antigen avoidance group underwent all sampling except peripheral lung biopsies. ^†^For horses with unknown history, disease duration was estimated as starting one year before the moment they joined the research herd, a possible underestimation. ^¶^Disease severity was determined based on the horse clinical history and lung function data, with horses reaching at least once in the observation period in our research herd prior or during the study a respiratory resistance (R_L_) > 3 cmH_2_O/L/sec during disease exacerbations classed as those with increased disease severity.

### Lung function

**Figure 1:**
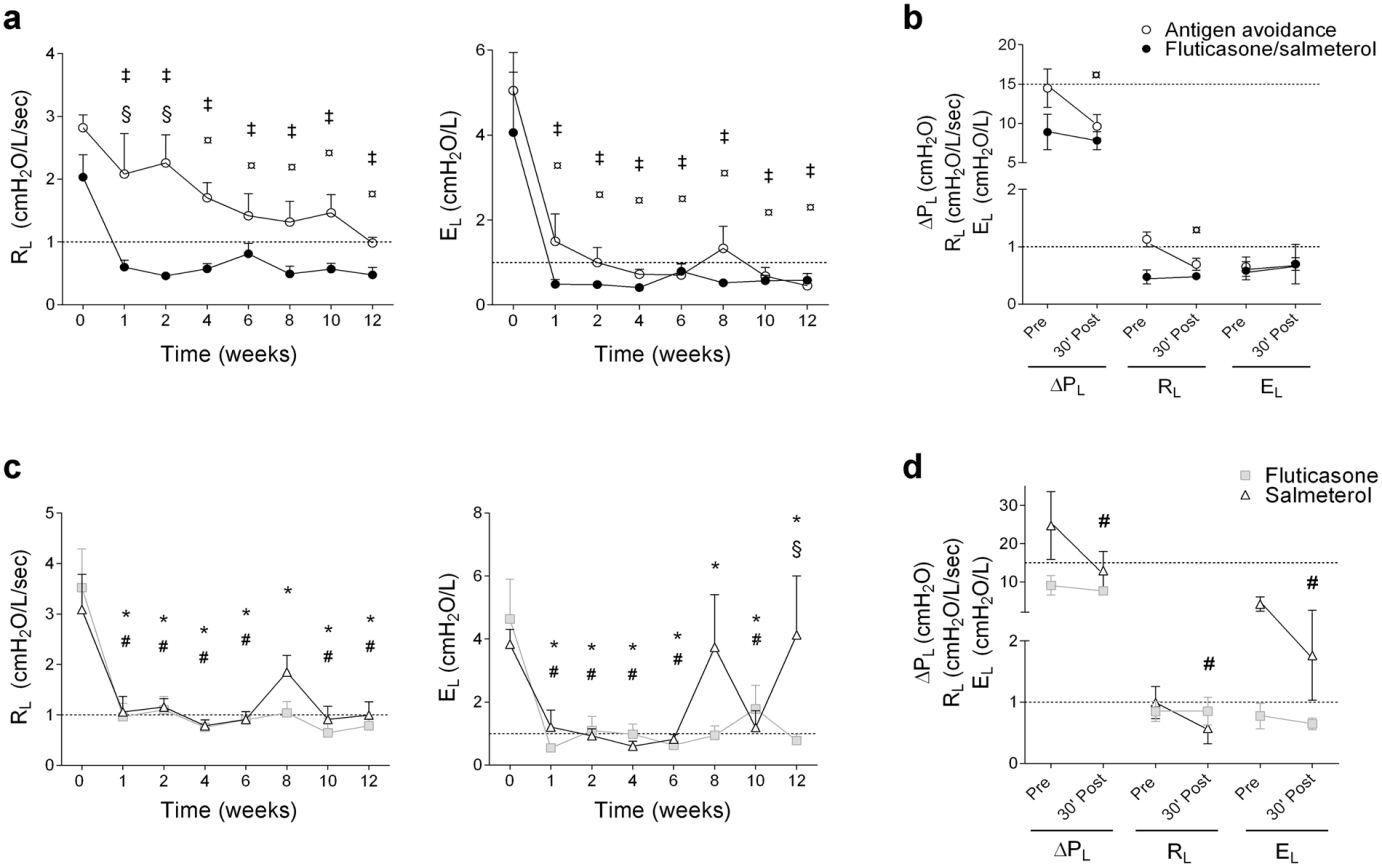
Pulmonary function and bronchoreversibility tests performed in study I (**a,b**) and II (**c,d**). Bronchodilator response was evaluated before and 30 minutes after the administration of inhaled albuterol 500 μg (**b**) or intravenous N-butyl-scopolamine 0.03 mg/kg (**d**) to detect residual airway obstruction. Values are expressed as means ± S.E.M. Dashed lines represent normal threshold in healthy horses. R_L_: pulmonary resistance; E_L_: pulmonary elastance; ΔP_L_: swing in transpulmonary pressure. ‡different from baseline of the same group for fluticasone/salmeterol (p < 0.0001), ¤different from baseline for antigen avoidance (p < 0.05), *different from baseline for fluticasone (p < 0.0001), #different from baseline for salmeterol (p < 0.0001), §difference between groups at the time point indicated (p < 0.05).

At baseline, all horses had severe airway obstruction as demonstrated by their increased pulmonary resistance and elastance compared to normal values (Fig. 1a,c). Fluticasone/salmeterol normalized pulmonary resistance and elastance from week 1 to 12. Antigen avoidance induced a similar rapid decrease in elastance, while resistance decreased only after 4 weeks and remained above normal values in 4/7 horses at 12 weeks (Fig. 1a). Fluticasone and salmeterol administered separately both decreased resistance and elastance after 1 week of treatment, which was maintained until week 12 only by fluticasone. The effect of salmeterol on pulmonary function was partly lost in 3 out of 6 horses after 8 weeks of treatment. It was only temporarily restored by a single dose of oral dexamethasone (0.06 mg/kg) administered at the end of week 8^21^ (Fig. 1c). At week 12, residual bronchoconstriction was detected in antigen avoidance and salmeterol-treated horses (Fig. 1b,d).

### Airway luminal inflammation

**Figure 2:**
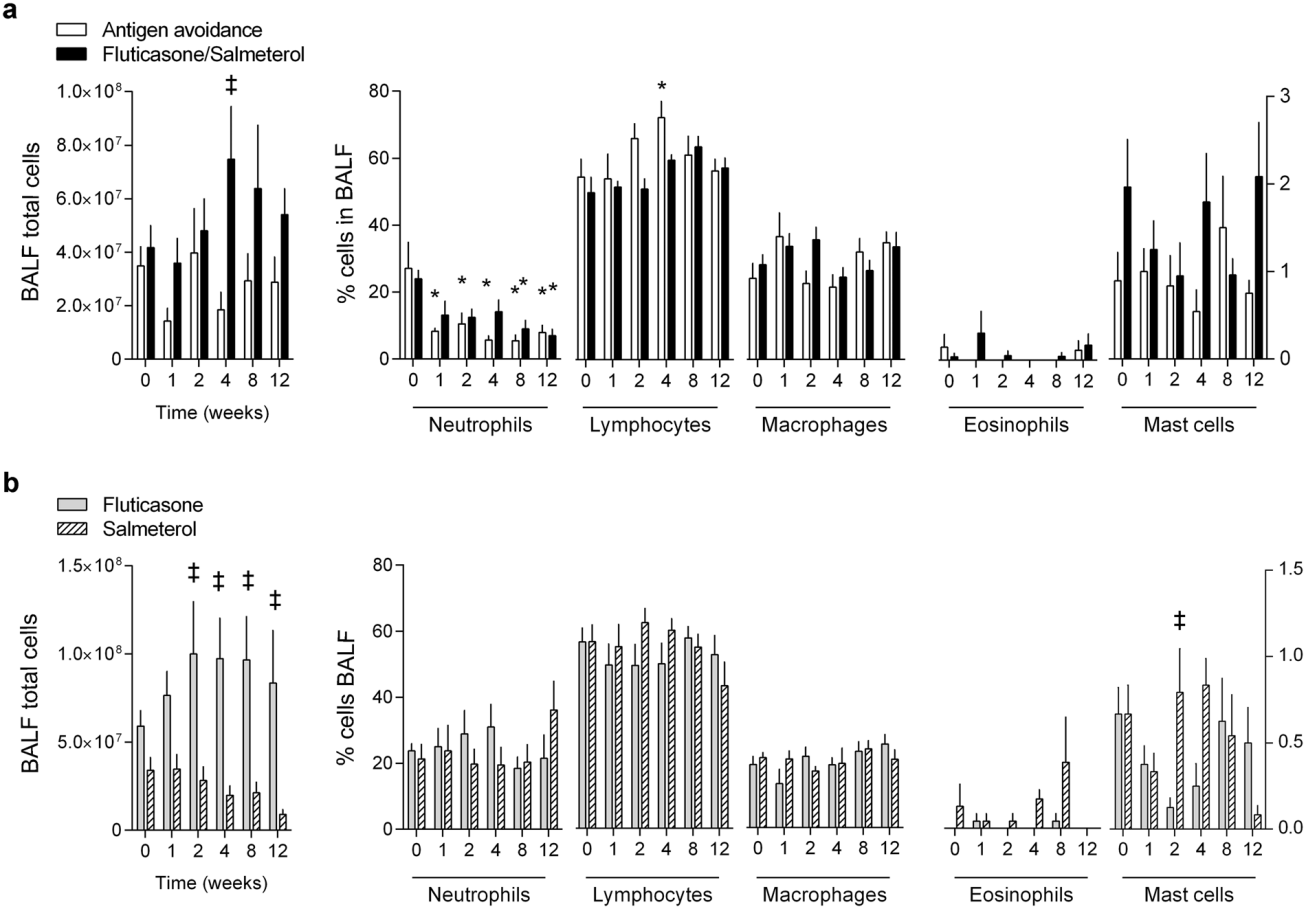
Bronchoalveolar lavage fluid cytology of study I (**a**) and II (**b**). Values are expressed as means ± S.E.M. *different from baseline of the same group (p < 0.05); ‡Difference between groups at the time point indicated (p < 0.05). BALF: bronchoalveolar lavage fluid cytology.

Antigen exposure induced BAL fluid (BALF) neutrophilia (>20%) in all groups at baseline. Antigen avoidance reduced neutrophilia after 1 week, but only 3/7 horses had normal values (<5%) by week 12. Despite ongoing antigen exposure, fluticasone/salmeterol reduced BALF neutrophilia starting from week 8, with 3/6 horses reaching normal values at week 12 (Fig. 2a). Fluticasone and salmeterol monotherapies were ineffective (Fig. 2b).

### Peripheral remodelling

Peripheral remodelling was assessed in 10 ± 4 airways/horse/time during study I and 10 ± 3 during study II (range: 3–25). Average airway Pi was 811 ± 221 μm in study I and 883 ± 456 μm in study II (range: 160–1996 μm). It was similar between groups and time points studied.

**Figure 3:**
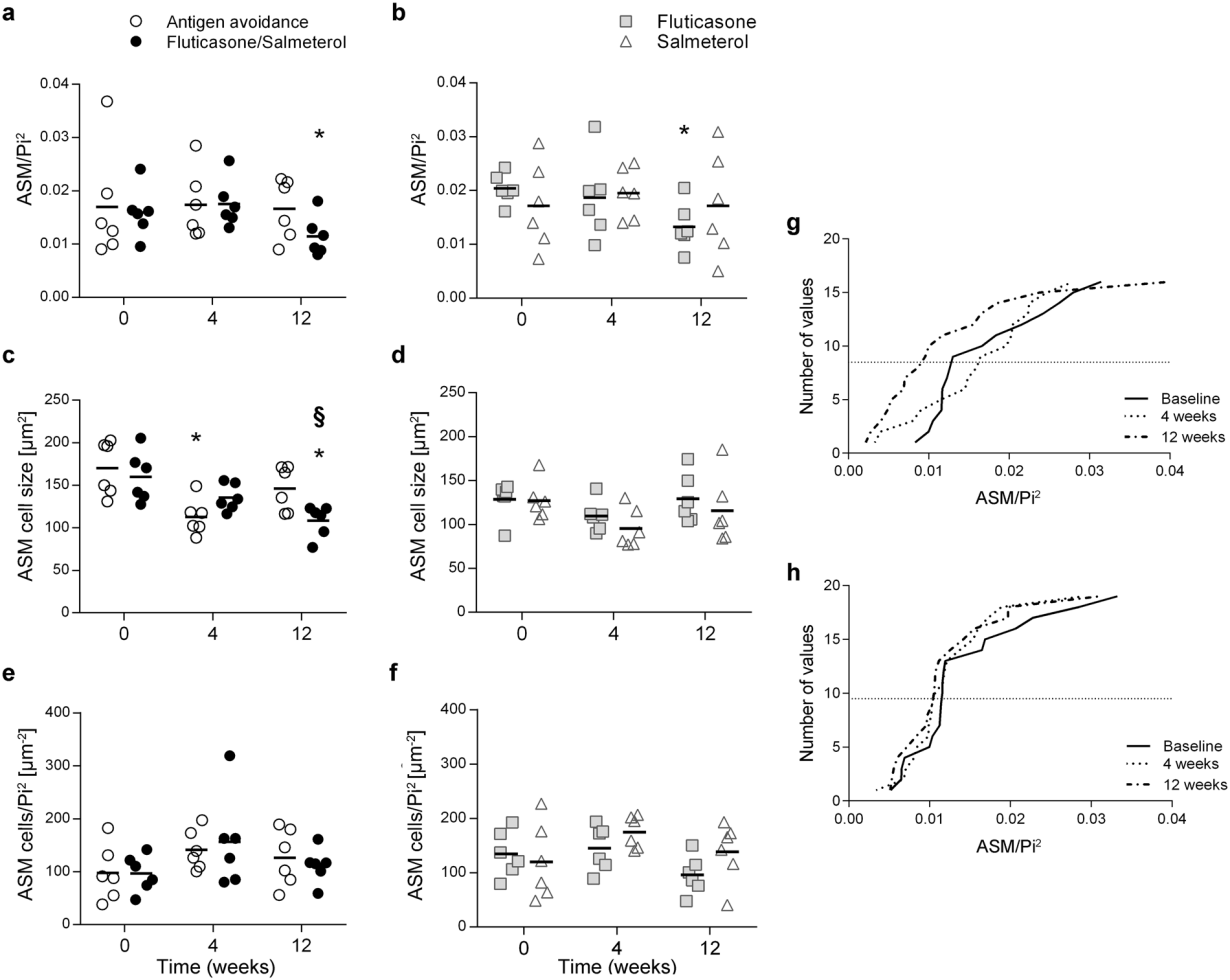
Peripheral airway smooth muscle remodelling. The corrected quantity of ASM, as well as ASM cell hypertrophy and hyperplasia were assessed by histology in study I (**a,c**, and **e**, respectively) and II (**b,d**, and **f**). Each symbol represents one horse (mean value of multiple measures performed). *Different from baseline of the same group (p < 0.05); §difference between groups at the time point indicated (p < 0.05). ASM: airway smooth muscle; Pi: internal perimeter of the airway. (**g,h**) Representative examples of the cumulative frequency distribution of peripheral ASM remodelling (ASM/Pi^2^) in one horse treated with fluticasone/salmeterol (**g**: n = 16 airways/time) and another one treated with antigen avoidance (**h**: n = 19 airways/time). Dashed lines identify median values.

At baseline, the ASM mass was similar in all treatment groups (Fig. 3a,b). It was comparable to previously reported values of ASM remodelling in asthmatic horses^22–24^. In the peripheral ASM layer, ECM was mainly comprised of elastin with rare collagen deposition (Supplementary Table S1). Overall, peripheral ASM mass was greater in asthmatic horses with a history of R_L_ > 3 cmH_2_O/L/sec, independently of treatment or time (p = 0.03, Supplementary Fig. S1). On average, fluticasone/salmeterol reduced ASM mass by 27% (range: 3–45%) at 12 weeks when compared to baseline (p = 0.007, Fig. 3a). Antigen avoidance had no effect. Inhaled fluticasone, but not salmeterol, reversed ASM remodelling at 12 weeks (p = 0.02, average reduction 33.6%, range: 4–62%, Fig. 3b) to the same extent observed with fluticasone/salmeterol.

**Figure 4:**
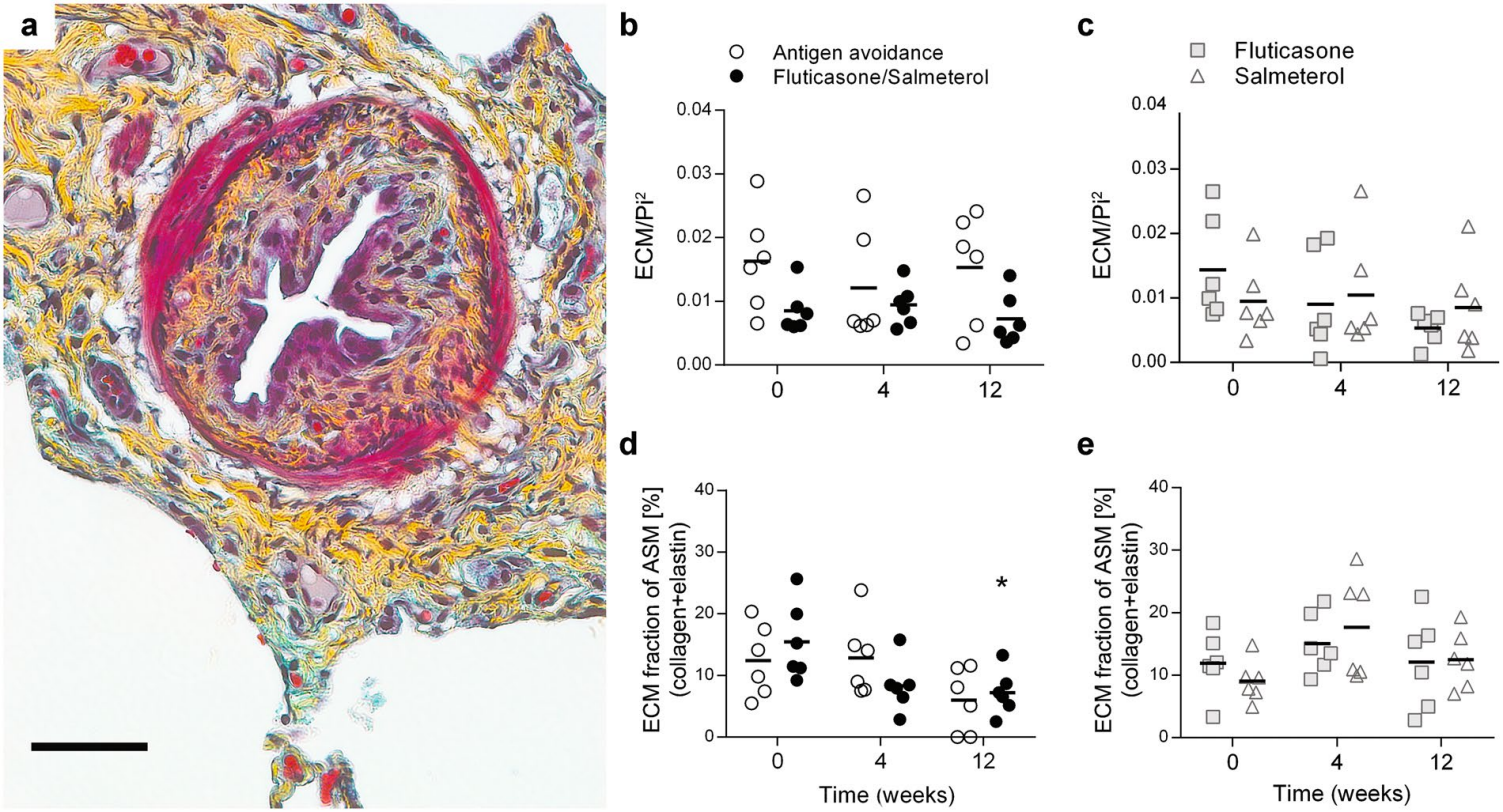
Peripheral ECM remodelling. (**a**) Representative image of a small airway of an asthmatic horse in which smooth muscle stains dark pink, collagen stains yellow, and elastin stains black (Russell-Movat pentachrome staining). Scale bar: 50 μm. (**b,c**) Corrected area of total ECM within the lamina propria of small airways of the horses participating in study I (**b**) and II (**b**). Each symbol represents one horse (mean value of multiple measures performed). (**d-e**) ECM fraction within the peripheral ASM layer of horses participating in study I (**d**) and II (**e**). *Different from baseline of the same group (p < 0.05). ECM: extracellular matrix; EF: elastic fibers; Pi: internal perimeter.

The number of myocyte nuclei/Pi^2^ remained unvaried along the study period in all groups (Fig. 3e,f). Myocyte size (Fig. 3c) and ECM fraction of ASM (Fig. 4d) were decreased by fluticasone/salmeterol, contributing respectively to 80% and 20% of the observed reduction of peripheral ASM mass at 12 weeks. Antigen avoidance only transiently decreased myocyte size at 4 weeks (Fig. 3c). Fluticasone did not affect myocyte size or ECM fraction of the ASM layer (Figs 3d and 4e). The normalized quantity of ECM in the lamina propria of peripheral airways was unaffected by 12 weeks of either treatment studied (Fig. 4b,c). Additional data on peripheral ECM fraction remodelling reversal are provided online (Supplementary Fig. S2).

In both studies, ASM mass correlated with myocyte nuclei/Pi^2^ at baseline (r = 0.92, p < 0.0001 in study I; r = 0.87, p = 0.0003 in study II) and after 12 weeks (r = 0.87, p = 0.0002 in study I; r = 0.8, p = 0.001 in study II), whereas after 4 weeks of treatment the correlation was significant only for fluticasone-treated horses (r = 0.85, p = 0.03). Values of ASM mass at baseline and at 12 weeks were correlated in fluticasone/salmeterol (r = 0.84, p = 0.03), antigen avoidance (r = 0.85, p = 0.03), and salmeterol (r = 0.81, p = 0.048), but not in fluticasone-treated horses (r = −0.07, p = 0.9).

### Central remodelling

On average, 5 EBBs/horse/time were analysed in both studies (range: 3–6), of which 3.5 ± 1.8 considered of good-to-optimal quality in study I and 3.8 ± 1.4 in study II, with no differences between groups and time points studied.

**Figure 5:**
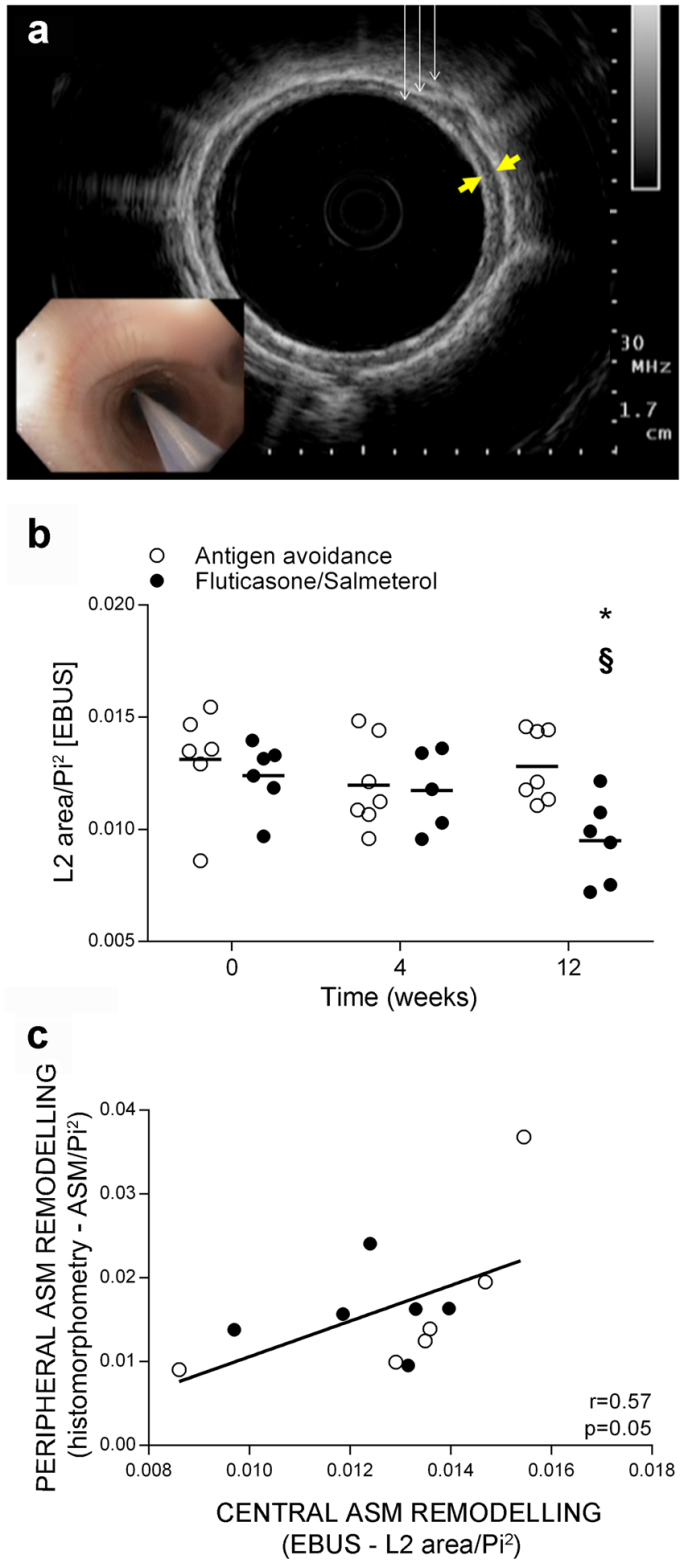
Assessment of central airway remodelling with EBUS. (**a**) Representative image of a central airway obtained with EBUS in a horse with asthma. Thin white arrows indicate the 3 hyperechoic layers of the bronchial wall representing, from the airway lumen going outwards, the bronchial epithelium, the inner, and the outer borders of the bronchial cartilage. The distance between the thick yellow arrows illustrates the thickness of L2. (**b**) EBUS-assessed central airway remodelling in study I. (**c**) Correlation between EBUS-assessed central airway remodelling and peripheral ASM remodelling assessed at histology at baseline (T0) in study I. Each symbol represents one horse (mean value of multiple measures performed). L2: second layer; EBUS: endobronchial ultrasound; ASM: airway smooth muscle; Pi: internal perimeter. *Different from baseline of the same group (p < 0.05); §difference between groups at the time point indicated (p < 0.05).

**Figure 6:**
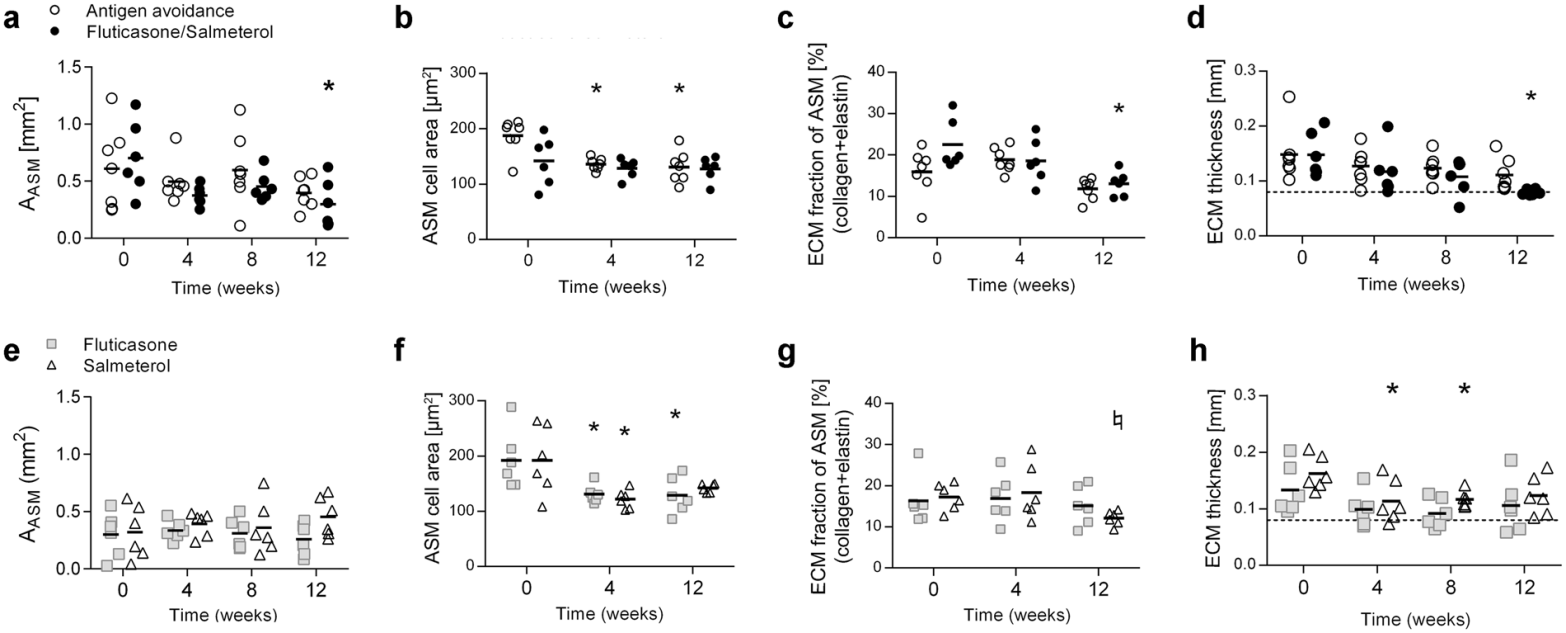
Central ASM and ECM remodelling. ASM area, myocyte size, ECM fraction of the ASM, and thickness of the ECM in the lamina propria were assessed in study I (**a**–**d**) and II (**e**–**h**). Each symbol represents one horse (mean value of multiple measures performed). Dashed line in panels **d** and **h** corresponds to the mean thickness of ECM reported in healthy horses in a previous study^19^. *Different from baseline of the same group (p < 0.05); ♮p = 0.05 from baseline of the same group. ASM: airway smooth muscle; ECM: extracellular matrix.

At baseline, EBUS yielded values similar to those previously published using this technique in asthmatic horses, indicating increased ASM mass^25^. Only fluticasone/salmeterol reduced submucosal remodelling as indicated by EBUS at 12 weeks (Fig. 5b), concomitantly with a reduction of the ECM fraction of central ASM (Fig. 6c) and of the lamina propria thickness, which reacquired values similar to those reported in healthy horses^19^ (Fig. 6d). This change was accompanied by a concomitant decrease in ASM area within endobronchial biopsies (Fig. 6a). Fluticasone monotherapy did not affect ASM area, ASM composition or lamina propria thickness. Salmeterol treatment did not alter ASM area (Fig. 6e), but it tended to reduce the ECM fraction of central ASM at 12 weeks (Fig. 6g) and partially decreased lamina propria thickness at 4 and 8 weeks, while its effect was lost at 12 weeks (Fig. 6h). Antigen avoidance did not affect central ECM. Further details on remodelling reversal of the central ECM fraction are available online (Supplementary Fig. S3). Myocytes of all groups reached a similar size at 4 weeks of treatment, and maintained this size until week 12. Myocyte size was significantly reduced by all treatments by week 4, but the effect was significant only for fluticasone and antigen avoidance treatment (Fig. 6b,f).

### Comparison between central and peripheral ASM

EBUS-assessed central ASM remodelling negatively correlated with endobronchial biopsy ASM area at baseline in all horses (r = −0.70, p = 0.01) and at 12 weeks only in fluticasone/salmeterol-treated horses (r = −0.85, p = 0.06). A negative correlation was expected as an increased thickness of the lamina propria could prevent a deep sampling within the smooth muscle layer^19^. EBUS-assessed central ASM remodelling also correlated with peripheral ASM mass (ASM/Pi^2^) at baseline in all horses (Fig. 5c) and at 12 weeks only in fluticasone/salmeterol-treated horses (r = 0.88, p = 0.03).

ASM bundle composition differed in peripheral and central airways, independently of treatment or time. Overall, ECM fraction was higher centrally than peripherally (p = 0.0002). Within the ECM fraction, collagen predominated centrally while elastin prevailed peripherally (p < 0.0001, Supplementary Table S1). A linear relationship was observed in study I between central and peripheral myocyte size (p = 0.007), independently of treatment or time.

### Disease evolution across the study period in horses participating in both studies

Seven horses participated in both studies, equally distributed between groups. In order to assess whether this introduced any bias in our analysis, we compared the baseline values of these 7 horses (*veterans*) during the 2 studies, as well as their baseline value during study II compared to the baseline values of the 5 remaining horses newly included in the study (*recruits*). Baseline lung function was similar in these subjects during both projects, and it was similar in veterans and recruits at baseline of study II (Supplementary Table S2).

Peripheral ASM and ECM mass (ASM/Pi^2^ and ECM/Pi^2^, respectively) of veterans were similar at baseline of both studies, indicating the absence of a cross-over effect of study I over study II. Also, ASM/Pi^2^ and ECM/Pi^2^ values at baseline of study II were similar in recruits and veterans (p = 0.5 and p = 0.2, respectively). In central airways, the ECM thickness of veterans was similar in study I and study II (p = 0.5) and between recruits and veterans at baseline of study II (p = 0.3). Data are provided in Supplementary Table S3. ASM layer composition of central and peripheral airways did not vary across the studies in veteran horses (p = 1 and p = 0.2, respectively) and it was similar between veterans and recruits at baseline of study II (p = 0.2 and p = 0.9, respectively, Supplementary Fig. S4).

## Discussion

Airway remodelling is a hallmark of asthma affecting both the ASM and the ECM of peripheral and central bronchi, and its reversibility following treatment is a matter of debate^1^. The specific anti-remodelling effects of currently employed asthma treatments on the peripheral airways are largely unknown. Our study examined reversibility of peripheral ASM and ECM airway remodelling following administration of fluticasone, salmeterol, or their combination in an equine model of neutrophilic asthma. We also investigated the effects of these drugs on central airways in order to understand whether any aspect of central remodelling is indicative of the structural changes occurring more distally within the bronchial tree. Notably, in order to outline the specific contribution of mechanical stress to bronchial remodelling we assessed the effects of salmeterol monotherapy in our study, which is precluded in human asthma due to safety concerns^12^. Our results indicate that the reversal of peripheral ASM remodelling is mainly influenced by corticosteroid-mediated mechanisms and that it is not accompanied by the reversal of airway luminal inflammation. Based on our data, the number and size of ASM cells are not affected by mechanical stress acting on the airways. Bronchospasm inhibition however reduced the quantity of bronchial ECM, both within and outside the ASM layer, with a greater effect observed centrally than peripherally.

Severe neutrophilic asthma represents an uncommon phenotype of the disease associated with peripheral airway dysfunction^10^. However, no studies have specifically described the structural changes affecting the peripheral airways of this subtype of patients or their response to treatment to date. In this perspective, severe equine asthma represents a unique model to study unknown aspect of the disease. Such as severe neutrophilic asthmatics, horses affected by severe equine asthma display bronchoalveolar neutrophilia and they are poorly responsive to low-dose ICS as reliever therapy, while they are effectively controlled with high-dose ICS or oral corticosteroids^24^. In the present study we have administered high-dose fluticasone or combined fluticasone/salmeterol as both relief and maintenance therapy, in order to assess whether these symptomatically efficacious treatments differed in terms of airway remodelling reversal and inflammation control. The molecular basis of the synergistic anti-inflammatory effect of ICS/LABA combinations has been documented *in vitro* ^11^, and some studies suggest that LABA may selectively inhibit IL-8 concentration in BAL fluid and neutrophil influx into asthmatic airways *in vivo* ^26–28^. As activated neutrophils secrete chemicals able to induce tissue damage and involved in airway remodelling^29^, an efficacious control of airway neutrophilia could enhance airway remodelling reversal. Our findings support the potential of fluticasone/salmeterol to reduce pulmonary neutrophilia in asthma. Assuming that neutrophilic inflammation control can be maintained over time by fluticasone/salmeterol, further studies extended to >3 months will have to understand whether it can also potentiate ASM remodelling reversal.

ASM is the main determinant of airway obstruction in asthma^5^. A marked increased in peripheral ASM mass has been associated with the more severe forms of the disease^3^. Our study is in agreement with this finding as peripheral ASM mass increased with disease severity at baseline. Moreover, our results indicate that chronically established peripheral ASM remodelling is reversible *in vivo*, although only partly, by 3 months of ICS treatment. The same degree of reversibility (~30% reduction) has been reported in asthmatic horses after 6 and 12 months of ICS treatment^24^, suggesting that components of peripheral ASM remodelling, once established, may be irreversible. This may be due to «premodelling» (congenital remodelling preceding symptoms)^30^ or to a disproportion between the detrimental effects of inflammation during the establishment of remodelling and the beneficial effects of inflammation control during remodelling reversal. A mathematical model of ASM growth in disease has shown that years of complete inflammation control may be required to counterbalance the ASM mass increase caused by a short period of severe airway inflammation^31^, whereas persistent mild-degree inflammation, as observed in asthma despite treatment^32, 33^, would freeze ASM mass in its remodeled state, preventing reversal. In either way, both the increased ASM and uncontrolled small airway dysfunction reported in asthmatic patients considered adequately treated^4, 8^ are in agreement with our findings. The absence of a significant decrease in ASM mass with salmeterol and the similar effect observed with fluticasone and fluticasone/salmeterol treatment on peripheral ASM remodelling reversal suggest that a corticosteroid (anti-inflammatory) treatment is necessary to reverse the increased ASM mass in asthma while mechanical stress *per se* is unlikely to influence ASM cell number and size. Fluticasone could have reduced the ASM mass via direct and indirect action on ASM cells. Corticosteroid administration suppresses ASM cell proliferation^34^ and modulates the genetic expression profile of ASM cells in asthmatic patients^35^ by increasing myocardin expression, a marker of ASM cells differentiation and contractility. In agreement with these findings, myocardin expression decreases in bronchial biopsies obtained after antigen challenge in asthmatic horses^36^. Recent data also support a neutrophil-mediated increase of ASM cell proliferation in severe equine asthma^37^. For this reason, the expected inhibitory effect of ICS on pulmonary neutrophilia could have contributed to the observed reduction in peripheral ASM mass in our study. Counterintuitively, however, airway luminal neutrophilia decreased only after 8 weeks of fluticasone/salmeterol treatment, while fluticasone monotherapy was ineffective. Although an increased risk of pneumonia is reported in asthmatic patients treated with inhaled fluticasone^38^, our horses did not show signs of pneumonia during the study period (fever, lethargy, pleurodynia), excluding the hypothesis that the blunted response observed in the fluticasone group could have been associated with infection. Further studies are warranted to clarify whether luminal neutrophilia reliably reflects peripheral bronchial wall and parenchymal inflammation in severe equine asthma.

Increased or altered ECM deposition occurs in the submucosa of asthmatic airways and contributes to airway remodelling in asthma^1^. Scientific evidence sustains a role, possibly synergic, for both inflammation and mechanical stress in the regulation of ECM deposition within the airways^2, 39^, but little is known concerning its reversal following treatment. We observed that the inhibition of ECM deposition differed across bronchial structures (lamina propria vs. ASM) and sites (peripheral vs. central). Overall, our data suggest that regulation of ECM deposition in severe equine asthma is mainly regulated by β-adrenergic-mediated mechanisms, although ICS could also contribute to it. Within the ASM layer, ECM deposition was reversed both peripherally and centrally by fluticasone/salmeterol. Salmeterol monotherapy also reduced the amount of collagen at both levels, although to a lesser degree than that observed with fluticasone/salmeterol. Contrarily, within the lamina propria, ECM deposition decreased only in central airways. A complete reversal was observed with fluticasone/salmeterol, while fluticasone and salmeterol monotherapies showed either a partial or transient effect, respectively. Intrinsic differences between central and peripheral fibroblasts^40^ may explain our findings. Alternatively, the presence of different inflammatory environments or mechanical forces acting on the lamina propria of central and peripheral airways, or the lower peripheral deposition of inhaled drugs could also have contributed, as high-doses of ICS are needed to reduce ECM deposition within the lamina propria in asthmatic patients^41^. In summary, our results sustain a significant effect of mechanical stress inhibition on ECM deposition at all levels of the bronchial tree, which is enhanced when concomitant corticosteroid-regulated mechanisms are activated. They also suggest that ECM deposition in the lamina propria of peripheral airways is less responsive to treatment than that of the central airways in neutrophilic asthma, although the reasons of these findings remain to be elucidated.

Different ECM components can modulate the mechanical behaviour of the airways during bronchoconstriction^42^. For this reason, there is growing interest in the composition of the ASM layer in asthma. Nevertheless, the composition of the ASM layer and its variability along the human bronchial tree has been investigated only in a limited number studies. In asthmatic horses, the ECM fraction of the ASM layer is mainly made up of elastin peripherally and of collagen centrally, which reflects the mechanical properties of the lungs. This is in agreement with a human study showing that the fractional area of the elastic fibers within the ASM is higher in the small than in the large airways of nonfatal asthmatics^43^. James and colleagues reported that, on average, 18–20% of the ASM layer is occupied by ECM both in central and peripheral airways of asthmatic patients^4^, which is also in line with our results. Yick and colleagues reported values of elastin and collagen fraction in the central airways of mild asthmatic subjects of 1–3% and 10–30%, respectively^44^, which closely resemble those we observed in the central airways of asthmatic horses. Finally, Araujo and colleagues reported an increased elastin fraction in central and peripheral ASM of fatal asthmatics (14–17%) compared to non-fatal asthmatics (2–3%) and healthy patients (7–8%)^43^. Overall, asthmatic horses appear to have elastin fractions in ASM similar to those reported in peripheral airways of fatal asthmatics and in central airways of mild asthmatics. Similarly, the collagen fraction observed in equine central airways is comparable to that observed in mild to moderate asthmatics. However, the collagen fraction of ASM in peripheral airways appears to be significantly lower in horses compared to human asthmatics. Whether this is due to intrinsic differences in the ASM cells or in the airway fibroblasts lying within the bronchial matrix, as shown in asthma^45^, or in both these cell-types, remains to be clarified. Besides providing evidence that a structural dichotomy exists between central and peripheral ASM composition in asthmatic horses, our results also highlight that it can be modulated by pharmacological treatment. Specifically, the combined administration of fluticasone and salmeterol is effective at reducing the elastin and collagen fraction of ASM in the peripheral and central airways, respectively. Of note, the collagen fraction of peripheral airways was also decreased in all subjects with fluticasone/salmeterol treatment (p = 0.04 with paired t-test) but it was not statistically significant after Bonferroni correction for multiple comparison. Interestingly, this effect was also observed in horses treated with salmeterol monotherapy but not in horses treated with fluticasone monotherapy, suggesting that β_2_-adrenoceptor-mediated mechanisms are likely to be involved. Similarly to our results, asthmatic patients who received regularly corticosteroids in addition to short-acting bronchodilators had a reduced quantity of elastin in their large and small airways^43^. Also, an increased ECM deposition has been reported in the central ASM layer of moderate asthmatics compared to severe asthmatics and control, which can be the consequence of the higher corticosteroid and bronchodilator regimen implemented in the latter group^46^. *In vitro* evidence exists supporting the ability of β_2_-adrenoceptor agonists at reducing pro-fibrotic events in human airways^47, 48^.

Peripheral airway assessment is rarely implemented in human respiratory research due to ethical considerations, as it requires invasive procedures to be performed^16^. Indirect evaluation of peripheral airway dysfunction in asthma is limited by the paucity of diagnostic tools available, and the need expressed by the respiratory community to develop non-invasive (or less invasive) markers of peripheral airway structure and function remains currently unmet^15^. One of the goals of our study was to assess whether elements of peripheral airway remodelling or their reversal could be predicted based on remodelling affecting the central airways. Our results suggest that, when EBUS is used to assess central airway remodelling and it is assumed that changes observed by EBUS proportionately reflect ASM dynamics^25^, the onset and extent of ASM reversal observed centrally reflects what occurs in the peripheral airways. Although EBUS is a complex procedure to be implemented, it is non-invasive and our data support its use for indirect peripheral airway structure assessment. Further studies, however, will have to confirm our findings. Despite a linear relationship was observed between myocyte size of central and peripheral ASM cells in Study I, it was not detected in Study II, suggesting it is not a constant finding.

During our study, salmeterol-treated horses experienced disease exacerbations after 8 weeks of treatment. Based on the results of a meta-analysis including more than 100 trials^49^, regular β-agonists use is now discouraged in people^12^ as it worsens asthma control and increases mortality. Our observations are in agreement with this finding. Despite effectively bronchodilating the airways even when administered for extended periods of time (especially partial agonists such as salmeterol), β_2_-agonists increase airway responsiveness^50, 51^. Moreover, these drugs induce vasodilation as well as bronchodilation, which could facilitate the influx of inflammatory cells into the airways or participate in airway wall thickening while masking clinical symptoms^52^. Based on our data, we cannot conclude about the effect of the treatments studied on the number of inflammatory cells into the airways, as the volume of the bronchoalveolar lavage recovered is variable and related to the degree of collapse of the airways in horses. However, horses treated with salmeterol monotherapy showed a blunted bronchodilator response to salmeterol treatment starting from week 9, which did not respond to a single administration of dexamethasone and thus which probably was not due to the occurrence of tachyphylaxis in ASM cells^21^. Also, remodelling parameters were unchanged at week 12 compared to the previous time points studied, suggesting that the reduced disease control was not related to a worsening of airway remodelling. Based on these observations, we postulate that the reduced disease control observed in horses might have been caused by an increased responsiveness of ASM cells to the antigenic stimulation. Alternatively, β_2_-agonist-induced goblet cell hyperplasia is described in murine models of asthma^53, 54^. The same mechanism, if proven to occur in asthmatic horses, could contribute to the increased airway obstruction we observed in our animals.

Several studies supports the fact that lung function decline is related to exacerbation frequencies^55–57^ and that it can be reduced by long term corticosteroid administration^58, 59^ in human asthmatic patients, while no similar data are available for asthmatic horses. As 7/18 animals participated in both studies, we compared the baseline data of these animals in the two studies in order to exclude the possibility of any cross-over effect. We found no significant differences in the lung function or remodelling parameters assessed. This finding is meaningful for two reasons: firstly, it suggests that established airway remodelling does not significantly worsen over a period of 1 year (3 months duration of study I + 8 months elapsing between study I and study II + 1 month of antigen exposure of study II before baseline values were collected), which is reflected by the stable lung function data in the same subjects. Secondly, it represents the first evidence of a “return to the original pathological remodelling” when treatment is stopped and horses are re-exposed to environmental triggers of asthma exacerbations. These observations, joined to the fact that the same degree of peripheral ASM remodelling (increased about threefold compared to control animals) was observed in asthmatic horses participating in different studies^22, 23^, suggest that the effect of chronic inflammation on ASM remodelling reaches a plateau early in disease development, and that such inflammatory milieu, if not constantly hindered, can quickly reactivate peripheral ASM and re-establish airway remodelling. Our data indicates that the significant 30% reduction observed in ASM mass after 12 weeks of ICS treatment is not maintained in time after the treatment is stopped, and such effect is completely lost after 8 months of ICS withdrawal. Further studies are required to better elucidate the kinetics of remodelling reestablishment after treatment withdrawal.

In conclusion, this study shows that at least 3 months are required to significantly decrease ASM mass in the equine model of neutrophilic asthma when fluticasone is administered at high dosage. Salmeterol add-on therapy allowed luminal neutrophilic inflammation control but did not enhance the reversibility of peripheral remodelling over fluticasone monotherapy. The mechanisms by which fluticasone reduces peripheral ASM mass remain to be elucidated although our data suggest that a reduction in ASM cell size is likely to be implemented before ASM cell number is reduced. The reduction of ECM deposition within the ASM layer appears as a β-adrenergic mediated mechanism. In central airways, fluticasone/salmeterol completely reversed ECM remodelling, while their effect was blunted when administered as monotherapies. Overall, the observed effect of ICS/LABA on ECM remodelling and luminal inflammation could contribute to the improved clinical response observed in severe asthmatics; however, their similar effect on peripheral ASM may explain why complete disease remission is not achieved in these patients.

## Methods

### Experimental design

**Figure 7:**
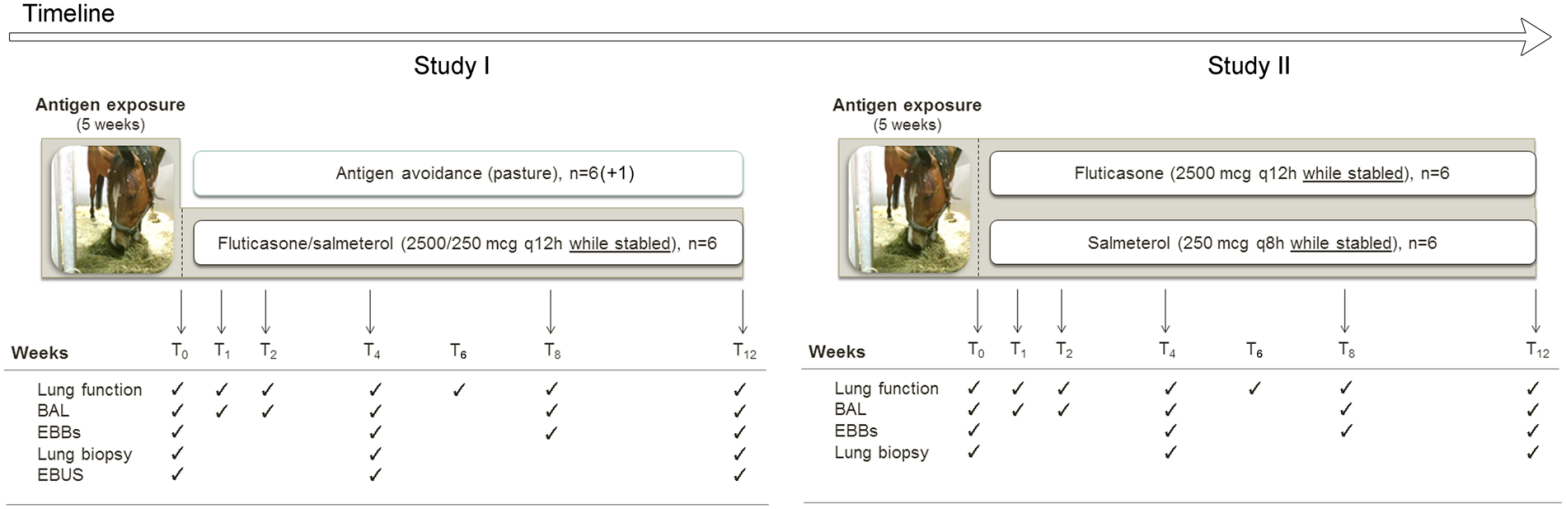
Study design. Grey background indicates antigen exposure. BAL: bronchoalveolar lavage; EBB: endobronchial biopsies; EBUS: endobronchial ultrasound.

Experiments were performed on 2 consecutive years. Eighteen asthmatic horses were studied. They underwent 5 weeks of antigen exposure consisting of stabling and hay-feeding in order to induce clinical exacerbation of the disease. Complete blood counts and blood biochemistry profiles were performed at baseline (T0) in order to exclude any respiratory infection. They were then treated for 12 weeks with one of four treatments. During the first year of experimentations (study I), horses were either moved to a low-antigenic environment (pasture, considered the gold standard approach for controlling airway inflammation in heaves^24^, n = 7) or administered inhaled fluticasone/salmeterol (Advair^®^ 250 HFA MDI, GlaxoSmithKline, 2500/250 μg q12 h, n = 6). During the second year (study II), horses were administered either inhaled fluticasone (Flovent^®^ HFA MDI, GlaxoSmithKline, 2500 μg q12 h, n = 6) or salmeterol (Sigma-Aldrich, 250 μg q8 h, n = 6). Seven horses participated in both experimental sessions. All drug dosages have previously been validated for use in severe equine asthma^24, 60^. Horses receiving pharmacological treatments were kept in the offending environment (stable and hay diet) while treated. Pulmonary function, bronchoalveolar lavage (BAL), EBBs, peripheral lung biopsies, and endobronchial ultrasound (EBUS) were performed as described in Fig. 7. All the experiments described were conducted in compliance with the guidelines of the Canadian Council on Animal Care. Ethical reasons also prevented the inclusion of a placebo group. The study protocol was approved by the local Ethics Committee (Rech-1324).

### Drug administration

Fluticasone/salmeterol combination and fluticasone alone were administered by means of an equine aerosol chamber (AeroHippus, Trudell Medical International), following the manufacturer instructions. Salmeterol powder was solubilized in sterile PBS at a concentration of 250 μg/ml, using Tween20 (Sigma-Aldrich) to facilitate solubility^54^. The dose of salmeterol administered (250 μg q8 h) was supported by previous data^60^ and by the results of preliminary tests we realized on asthmatic horses showing a significant bronchodilator effect persisting up to 6 hours post administration (Supplementary Fig. S5). Salmeterol was administered using a face-tight mask equipped with a mobile ultrasonic nebulizer (SaHoMa, NEBU-TEC International, Germany).

### Lung function

Lung function was assessed as previously described^61^. Briefly, transpulmonary pressure was estimated by means of an oesophageal balloon and respiratory flows detected using a heated pneumotachograph connected to a face-tight mask placed on the horse’s nose. Pulmonary resistance and reactance were then computed using dedicated software (Labdat/Anadat program on MS-DOS, and flexiWare software, SCIREQ, Canada). A single-compartment linear model of the lung was employed, expressed as: P_L_ = (E_L_ × V_T_) + (R_L_×$\dot{{\rm{V}}}$) + k, where P_L_: transpulmonary pressure, E_L_: pulmonary elastance, V_T_: tidal volume, R_L_: pulmonary resistance, $\dot{{\rm{V}}}$: respiratory flow, and k: transpulmonary end-expiratory pressure. Residual bronchoconstriction was assessed at the end of the treatment period using inhaled albuterol (500 μg, in study I) or N-butyl-scopolamine bromide (0.3 mg/kg IV, in study II) at dosages previously demonstrated to be effective in asthmatic horses^62, 63^. In order to assess residual bronchoconstriction, lung mechanics were repeated before and 20 to 30 minutes after bronchodilator administration.

### Bronchoscopy

Bronchoscopies were performed on standing sedated horses (detomidine/butorphanol administered IV at 0.015/0.015 mg/kg). BALF was first obtained from a lung, randomly chosen for each animal at baseline and then systemically changed at every time point, stored on ice and processed within 2 hours for cytology assessment^64^. Differential cell counts were performed on a minimum of 400 cells on Wright-Giemsa stained cytospins of non-filtered BALF aliquots of 400 μm each. When cell density was too high to allow reliable counting, cytospins of 200 μm aliquots were prepared. Six to eight EBBs were obtained from the contralateral lung as previously described^19^, fixed in PFA for 24 hours, and paraffin-embedded. Care was taken to avoid biopsying twice the same bronchial bifurcation. EBUS images were obtained from the lung used for the BAL procedure following a protocol previously developed and validated in our laboratory^25^. Briefly, a 30 MHz radial miniprobe was employed (UM-S30-25R, Olympus,Richmond Hill, ON, Canada) with its dedicated balloon-ended sheath (MH-246R, Olympus,Richmond Hill, ON, Canada). Three to 5 images per airway were obtained from 8 to 10 bronchi ranging from 4 to 12 mm in diameter. The area of the second layer (L2) and the internal perimeter (Pi) were measured on digital images by the same observer blinded to the subject ID and L2 area/Pi^2^ calculated.

### Thoracoscopy

Thoracoscopies were performed on standing sedated horses (detomidine/butorphanol 0.015/0.015 mg/kg IV), restrained in a stock. Large peripheral lung biopsies (>5 cm^3^) were obtained by means of a cautery device (Ligasure, Covidien) and endoscopic staplers (Endo GIA, Covidien) as described in previous reports^65, 66^, fixed in PFA for 72 hours, and paraffin-embedded.

### Histomorphometry

Morphometry was performed on Russell-Movat-stained tissues^67^ using ImageJ (NIH, Bethesda, USA) and newCAST (Visiopharm, Hoersholm, Denmark). Preliminary studies comparing histomorphometry results obtained on Russel-Movat trichrome and immunostained tissues revealed a good agreement between the two methods (Supplementary Fig. S6). EBBs of good to optimal quality^19^ and peripheral bronchi <2 mm in diameter with a major to minor axis ratio < 1.5, ASM surrounding at least 70% of their circumference, and intact epithelium were studied at 40x magnification. In peripheral lung biopsies, the area occupied by ASM and extracellular matrix (ECM) was measured manually tracing the border of the ASM bundles (for ASM) and tracing the area enclosed between the basal membrane and the ASM layer (for ECM). This was possible as in peripheral airways this tissue is dense and clearly stains yellow (collagen), blue (proteoglycans), or black (elastic fibers, EF)(Fig. 4a). The area occupied by EF was estimated by point counting, using a grid superimposed to the image where each point corresponded to an area of 88 μm^2^. Total EF area was then calculated as (n points × 88 μm^2^). The number of myocyte nuclei within the entire ASM bundles of each airway was counted manually. The basal membrane length (Pi) was traced manually and used to correct the previously described variables for variation in airway size^25^. ASM bundle composition (fraction occupied by myocytes or ECM) was assessed by point counting, each point corresponding to an area of 88 μm^2^. ASM cell size was indirectly calculated (ASM area x ASM myocyte fraction/myocyte nuclei). In EBBs, ASM area, ASM composition, ASM cell size, and lamina propria thickness (epithelium-to-ASM distance) were assessed as previously described^19^.

### Statistical analysis

Statistical analysis was performed with SAS v.9.3 (Cary, NC, USA). Studies I and II were analyzed separately as 7 horses participated in both studies. Data distribution was assessed using the Kolmogorov–Smirnov test. Data not normally distributed were log 10 transformed. Unless otherwise stated, data are expressed as mean ± S.D. A repeated-measures linear model was employed with “time” as within-subject factor, “treatment” as a between-subjects factor. *A priori* contrasts with Benjamini-Hochberg correction were used for testing differences among groups and times (all time-points vs. baseline). “Disease severity” was defined based on R_L_ values. Horses presenting episodes of airflow obstruction with R_L_ > 3 cmH_2_O/L/s prior or during the study period were allocated to the group with increased disease severity. The relationship between disease severity and remodelling was assessed only at baseline to avoid cross-over effects with treatments. Paired t-test was used for analysis of residual bronchoconstriction after bronchodilator administration and peripheral ASM mass. A linear mixed model with “subject” as random-effect was used for studying associations between different parameters. Depending on data distribution, Spearman or Pearson test was used for correlations.

### Data availability

All data generated or analysed during this study are included in this published article (and its Supplementary Information files).

## Electronic supplementary material

Supplementary information
