# Supplementary material for: Fluticasone/salmeterol reduces remodelling and neutrophilic inflammation in severe equine asthma
Source: Sci Rep. 2017 Aug 18;7:8843. doi: 10.1038/s41598-017-09414-8 (PMC5562887; doi:10.1038/s41598-017-09414-8)
Supplement: Supplementary file 1 — Supplementary information [file 41598_2017_9414_MOESM1_ESM.pdf]

SUPPLEMENTARY INFORMATION

Fluticasone/salmeterol reduces remodelling and neutrophilic inflammation in severe equine asthma

Michela Bullone, Amandine Vargas, Yvonne Elce, James G. Martin, Jean-Pierre Lavoie.

RESULTS

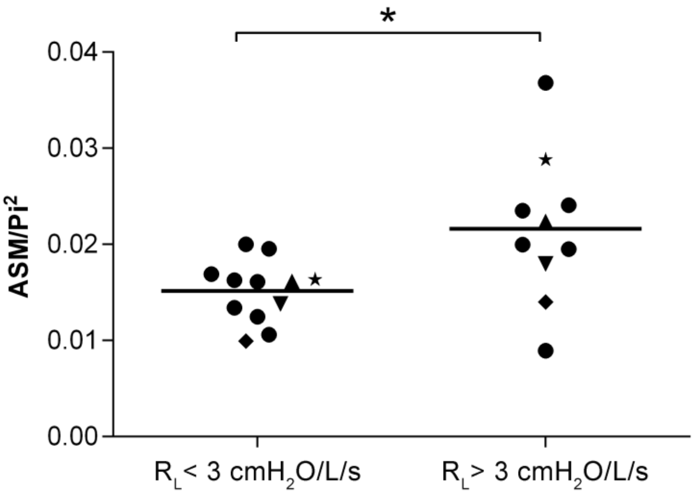

Supplementary Fig. 1. Effect of asthma severity on peripheral ASM remodelling during disease exacerbations (baseline). Four horses experienced episodes of airway obstruction with R_L>3 cmH2O/L/s in the time elapsing between the end of study I and the beginning of study II. For this reason, they have been classified differently in the two studies (indicated in the graph with non-circle symbols). Statistical analysis was performed with unpaired t test with Welch correction for unequal variances. Only baseline values (T0) were analysed to avoid any treatment-related bias. * : p=0.03.

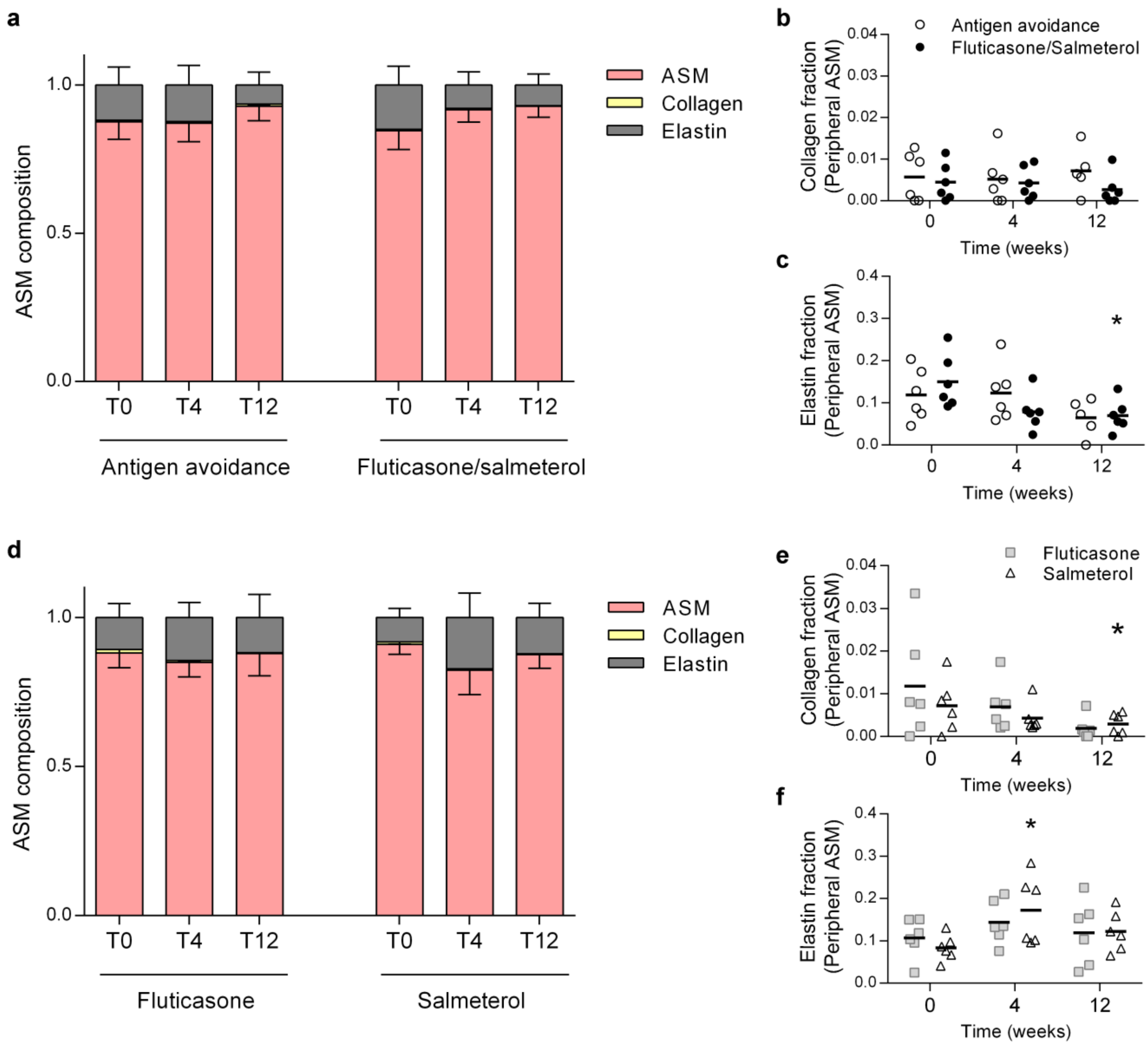

Supplementary Fig. S2. Treatment effects on ASM bundle of peripheral ASM bundles and effect of treatments. Panels a and d represents the total composition of peripheral ASM bundles in study I and II, respectively. Bars represent means ± S.E.M. The specific contribution of collagen and elastin is reported in panels b and c for study I, and in panels e and f for study II. Each point represents one horse (mean value of multiple measures). *: Different from baseline of the same group ($p<0.05$). ASM: airway smooth muscle.

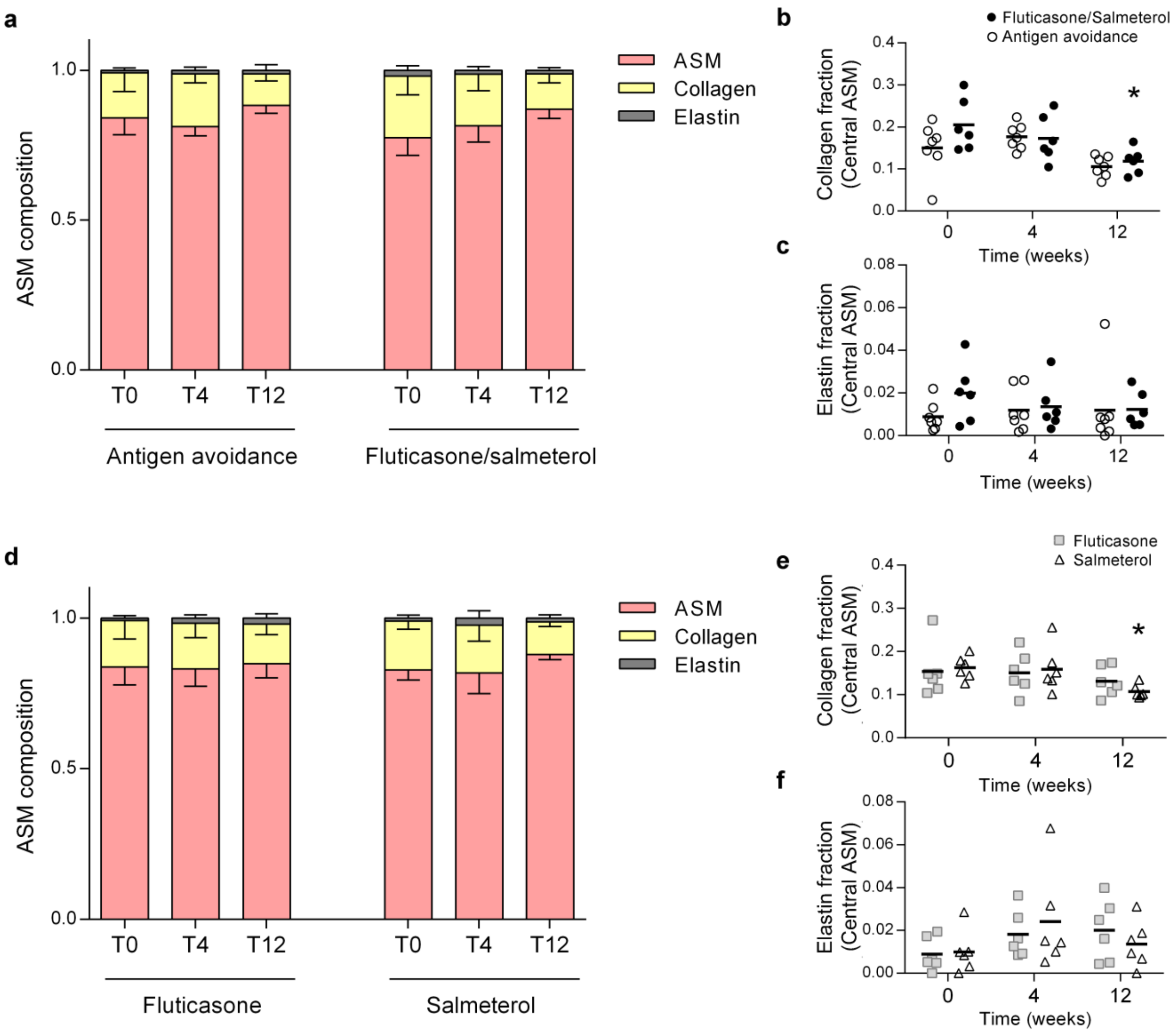

Supplementary Fig. S3. Treatment effects on ASM bundle composition of central airways. Panels a and d represents the total composition of central ASM bundles in study I and II, respectively. Bars represent means ± S.E.M. The specific contribution of collagen and elastin is reported in panels b and c for study I, and in panels e and f for study II. Each point represents one horse (mean value of multiple measures). *: Different from baseline of the same group ($p<0.05$). ASM: airway smooth muscle.

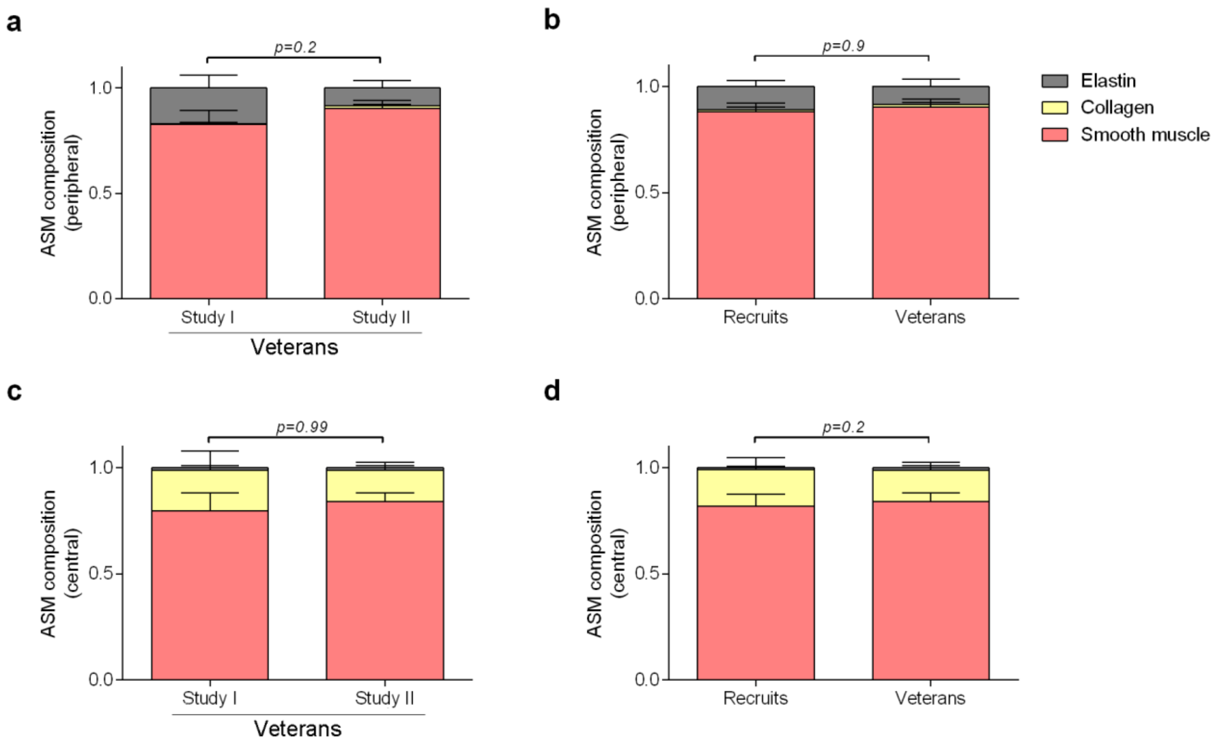

Supplementary Fig. S4. ASM composition evolution across the studies. ASM composition was similar in veteran horses at the baseline of study I and study II both peripherally (a) and centrally (c). In study II, ASM composition at baseline (T0) was similar in veterans and in recruits both peripherally (b) and centrally (d). Two-way ANOVA for repeated measures was used for statistical analysis. P values refer to the study effect (a,c) or to the group effect (b,d).

Supplementary Table S1. ASM bundle composition during severe equine asthma exacerbations (baseline).

	Peripheral ASM bundles			Central ASM bundles		
	Myocytes	Collagen	Elastin	Myocytes	Collagen	Elastin
Antigen avoidance	87±6	0.6±0.6	12±6	84±6	15±6	0.9±0.7
Fluticasone/Salmeterol	84±6	0.4±0.4	15±6	77±6	20±6	2±1.4
Fluticasone	88±5	1.2±1.2	11±5	84±6	15±6	0.9±0.8
Salmeterol	91±3	0.7±0.6	8±3	83±3	16±3	1±1

Values are expressed in percentage [%]as mean ± S.D. ASM: airway smooth muscle.

Supplementary Table S2. Disease evolution across the studies (lung function data).

	Veterans (study I)	Veterans (study II)	Recruits (study II)
ΔP_L [cmH_2O]	43.24 ± 21.09	48.15 ± 21.17	54.48 ± 24.04
R_L [$cmH_2O/L/s$]	2.641 ± 1.101	3.257 ± 1.719	3.666 ± 2.020
E_L [cmH_2O/L]	4.985 ± 3.431	3.402 ± 1.365	5.070 ± 3.096

Values are expressed as mean ± S.D. The values of Veterans (study I) and Veterans (study II) were compared with paired t-test. The values of Veterans (study II) and Recruits (study II) were compared with Mann-Whitney test.

Supplementary Table S3. Disease evolution across the studies (remodeling data).

	Veterans (study I)	Veterans (study II)	Recruits (study II)
ASM/Pi^2	0.0133 ± 0.0049	0.0172 ± 0.0049	0.0204 ± 0.0028
ECM/Pi^2	0.0093 ± 0.0042	0.0108 ± 0.0077	0.0087 ± 0.014
ECM thickness [mm]	0.149 ± 0.056	0.138 ± 0.033	0.161 ± 0.046

Values are expressed as mean ± S.D. The values of Veterans (study I) and Veterans (study II) were compared with paired t-test. The values of Veterans (study II) and Recruits (study II) were compared with Mann-Whitney test.

METHODS

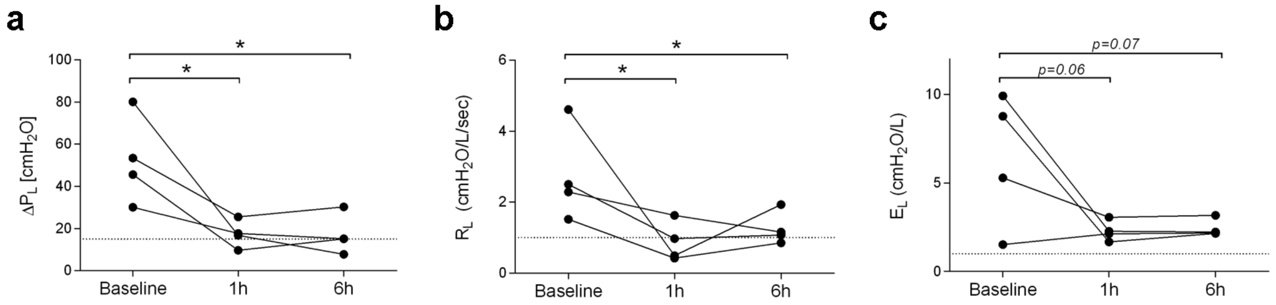

Supplementary Fig. S5. Preliminary study evaluating the effects of inhaled salmeterol (250 μg over 1 to 2 minutes period) to unsedated asthmatic horses. Transpulmonary pressure (a) and pulmonary resistance (b) were significantly reduced up to 6 hours post-administration. Although the effect on pulmonary elastance was not significant (c), it decreased in 3 out of 4 horses.

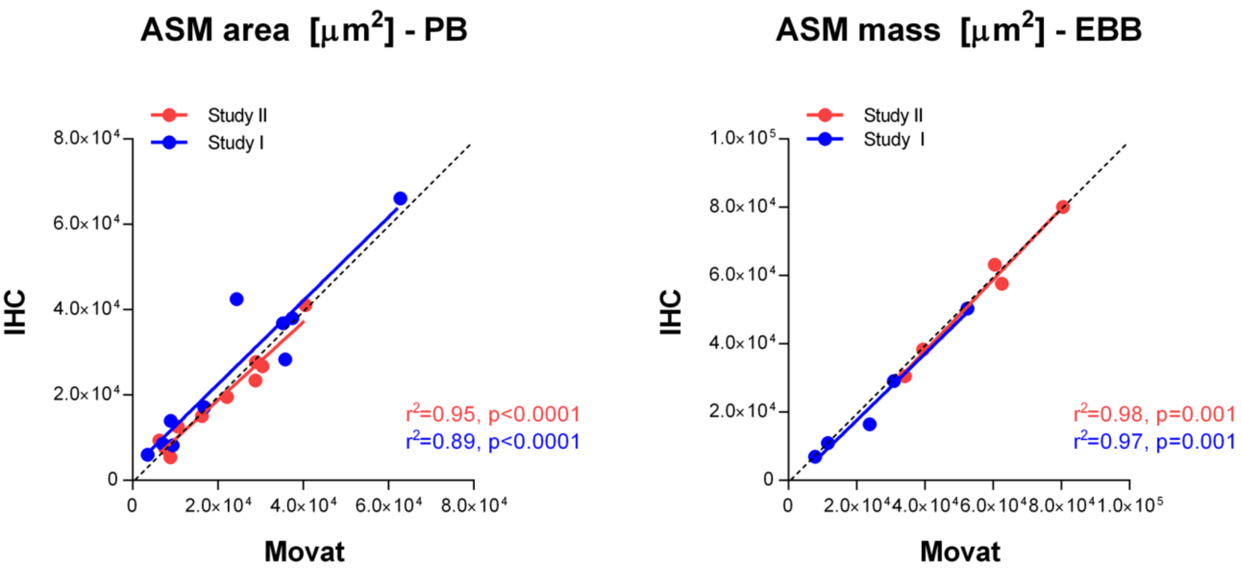

Supplementary Fig. S6. Preliminary study assessing the agreement between airway smooth muscle (ASM) histomorphometry measured on Russel-Movat trichrome stained tissues vs. immunostained tissues (anti-smooth muscle alpha actin, A2547, Sigma-Aldrich, USA). PB (pulmonary biopsy): each point corresponds to an airway. EBB (endobronchial biopsy): each point corresponds to a biopsy. IHC: immunohistochemistry.
